# Liquid Crystalline Block Copolymers for Advanced Applications: A Review

**DOI:** 10.3390/polym17182444

**Published:** 2025-09-09

**Authors:** Maryam Safari, Jules A. W. Harings

**Affiliations:** Aachen Maastricht Institute for Biobased Materials (AMIBM), Faculty of Science and Engineering, Maastricht University, Urmonderbaan 22, 6167RD Geleen, The Netherlands; jules.harings@maastrichtuniversity.nl

**Keywords:** liquid crystalline block copolymers, hierarchical structure

## Abstract

Liquid crystalline block copolymers (LCBCPs) have emerged as an adaptable hybrid class at the intersection of self-assembling block copolymers and liquid crystalline ordering, producing multi-tiered architectures that can be finely programmed for multifunctional performance. This review surveys recent advances in their structure–property relationships and highlights applications spanning nanotechnology, biomedical systems, flexible photonics, stimuli-responsive, energy storage, and soft robotics. Particular emphasis is placed on how molecular design enables precise tuning of structural, optical, mechanical, and stimuli-responsive functions, positioning LCBCPs as strong candidates for next-generation functional materials. We also discuss current challenges, including scalability, phase control, and advanced characterization, and outline promising research directions to accelerate their translation from laboratory concepts to real-world technologies.

## 1. Introduction

Liquid crystals (LCs) constitute a mesophase—an intermediate state of matter that bridges the characteristics of conventional liquids and crystalline solids. They flow like liquids, yet maintain a degree of molecular alignment reminiscent of solids [1,2,3,4]. Unlike ordinary fluids (exhibiting only short-range disorder) or crystals (with long-range periodic order), LCs preserve orientational order at mesoscopic scales, giving rise to unique anisotropic optical and mechanical properties [3,5,6]. This partial molecular alignment, typically along a preferred direction known as the director, underpins their anisotropic behavior and high responsiveness to external stimuli such as temperature, electric and magnetic fields, or light [2,5,7]. As soft matter, LCs are governed by intermolecular interactions comparable to thermal energy, facilitating abundant structural fluctuations at mesoscopic scales [5,8,9]. These dynamic structures enable LCs to exhibit a broad range of stimuli-responsive functions, which have been effectively harnessed in functional applications [2,10,11,12,13].

Liquid crystal polymers (LCPs) advance the capabilities of LCs by incorporating mesogenic units into polymeric architectures. This design fuses the fluidity and responsiveness of liquid crystals with the mechanical strength and processability of polymers [14,15,16,17]. Based on where the mesogenic units are attached, LCPs can be categorized as main-chain liquid crystal polymers (where mesogens are embedded in the polymer backbone) or side-chain liquid crystal polymers (where mesogens are pendant along a flexible spacer) [18,19,20]. Such structural versatility enables precise tuning of phase transitions, ordering, and functional behaviors, opening pathways to applications including responsive sensors, electromechanical actuators, aerospace fibers, and advanced composites [21,22,23,24,25,26,27].

**Scheme 1:**
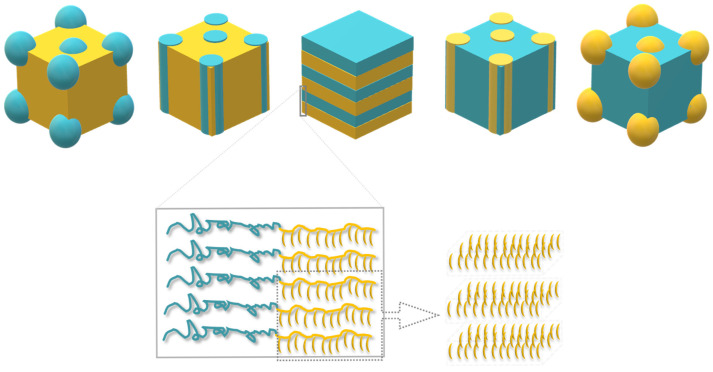
The exceptional nature of LCBCPs comes from their molecular architecture that couples self-assembling polymer blocks with sidechain liquid crystalline mesogens.

Building on these foundations, liquid crystalline block copolymers (LCBCPs) combine two powerful ordering principles: the microphase separation of block copolymers [28,29,30,31,32,33,34,35,36,37,38,39] and the anisotropic molecular ordering of liquid crystals [2,15,26,40]. In these hybrid macromolecules, chemically distinct polymer blocks—typically one mesogen-containing and the other flexible or amorphous—segregate into nanoscale domains such as lamellae, cylinders, gyroids, or spheres, while mesogenic segments within those domains form ordered LC phases, including nematic, smectic, or cholesteric arrangements (see Scheme 1) [40,41,42,43,44,45,46,47,48,49,50,51,52,53]. This hierarchical organization, spanning nanometers to micrometers, can be tuned by adjusting block volume fraction, mesogen chemistry, and spacer flexibility [54,55,56], enabling precise control over both nanoscale morphology and mesogen alignment [53,57,58].

Crucially, the coupling between microphase structure and LC ordering imparts dynamic responsiveness, schematically drawn in Scheme 1: variations in temperature, applied electric or magnetic fields, or light irradiation can reversibly reconfigure both the internal nanostructure and macroscopic behavior in real time [21,41,48,59,60,61,62,63,64,65,66,67,68,69,70]. This combination of tunable multiscale organization, anisotropic functional properties, and adaptive response is rarely achievable in conventional block copolymers or LCPs alone, underscoring the pivotal role of molecular architecture in defining LCBCP performance.

This review paper focuses on publications from peer-reviewed journals, primarily from the last decade (2016–2025), centered around essential keywords: “liquid crystalline block copolymer”, “LCBCP”, “block copolymer self-assembly”, and “liquid crystalline polymers”.

## 2. Recent Research Fields in LCBCPs

LCBCPs have emerged as a dynamic class of materials, attracting growing interest across diverse research fields due to their unique combination of self-assembly, anisotropic ordering, and stimuli-responsive function. This section highlights recent progress in harnessing LCBCPs for applications spanning nanotechnology, photonics, biomedicine, advanced functional materials, and energy storage, emphasizing their fundamental roles and transformative potential in these domains.

### 2.1. Nanostructured Materials and Nanotechnology

In nanotechnology, LCBCPs function as highly effective and precise templates for the fabrication of nanostructured materials. Their ability to self-assemble into well-defined morphologies (such as cylindrical, lamellar, or spherical domains) makes them an ideal case for creating ordered nanoscale architectures. These well-defined domains serve as templates for the synthesis of nanoporous membranes and nanostructured inorganic materials through selective etching or infiltration techniques [46,71].

For instance, Houben et al. created mechanically robust, aligned nanoporous smectic LC polymer networks within an anisotropic microporous scaffold. Post-polymerization, the composite membranes featured aligned 2D nanopores capable of rejecting anionic solutes above 322 g/mol, demonstrating their potential for precise molecular sieving

**Figure 1:**
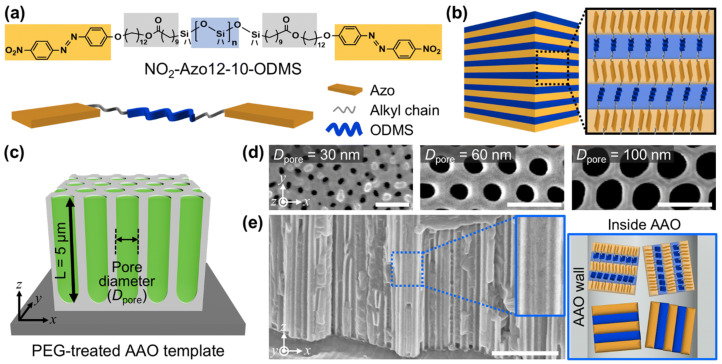
(**a**) Chemical structure and schematic illustration of NO2-Azo12-10-ODMS (Azo-LCBCO). (**b**) Schematic illustration of the lamellar superstructure comprising smectic layers formed via self-assembly of Azo-LCBCO. (**c**) Schematic illustration of PEG-treated anodic aluminum oxide (AAO) templates filled with Azo-LCBCO. (**d**) Top-down SEM (Scanning Electron Microscopy) images of AAO templates with pore diameters (D_pore_) of 30, 60, and 100 nm. Scale bars are 200 nm. (**e**) Cross-sectional SEM image of an AAO template with a D_pore_ of 60 nm filled with Azo-LCBCO, along with a schematic illustration of the self-assembled lamellar structures within the AAO nanochannel. The scale bar is 500 nm. The figure was reproduced with permission from Ref. [72].

Structural control can be further refined through confinement strategies. In a recent study, Kim et al. [72] examined how nanoconfinement influences the self-assembly and alignment of liquid crystalline block co-oligomers (Azo-LCBCO) within anodic aluminum oxide (AAO) templates (see Figure 1). Using GI-SAXS, they found that reducing pore diameter from 100 nm to 30 nm not only promoted bilayer formation but also introduced interdigitated and monolayer smectic phases, revealing greater structural diversity in tighter confinement. GI-WAXD analysis showed that the proportion of smectic layers aligned parallel to the pore axis increased dramatically from 22% to 93.7%, supported by azimuthal angle evaluation of π–π stacking. These findings demonstrate that nanoscale confinement offers precise control over smectic polymorphism and molecular orientation, providing a versatile strategy for engineering nanostructured soft materials with tunable anisotropic properties.

Parallel to the influence of confinement, compositional design strategies have also been used to tune nanostructure and functional performance in LCBCPs. In a recent study, Zhou et al. [73] synthesized a block copolymer (BCP) combining an LC azobenzene polymer with an ionized polymeric ionic liquid, showing that its nanostructure changes with composition and temperature. Notably, the material’s ionic conductivity improved markedly at higher temperatures, highlighting its promise as a solid polymer electrolyte [73].

Similarly, targeted molecular design of LCBCPs can enable precise control over microphase-separated morphologies and LC alignment. In 2023, Takahashi et al. [74] synthesized novel smectic LC diblock copolymers, poly(methacrylate) featuring side chains terminated with cyano-functionalized phenyl benzoate mesogens, called PEO-*b*-PM6BACP, using atom transfer radical polymerization, ATRP. These BCPs featured sidechain cyano-terminated phenyl benzoate mesogens and formed hexagonally packed cylindrical microphase-separated structures with lattice periods around 18 nm. The LC behavior and molecular alignment within the PEO-*b*-PM6BACP system indicated precise control over morphology via LC incorporation in block frameworks [74].

In a striking demonstration of self-assembly’s precision, Luo et al. [75] reported a one-step assembly pathway in which a liquid crystalline block copolymer formed uniform cylindrical micelles that spontaneously coupled end-to-end into hierarchical supramicellar nanofibrils. By tuning nucleation and aggregation tendencies through controlled assembly conditions, the length of individual cylinders and the overall fibril morphology could be precisely adjusted. The liquid-crystalline ordering of the micellar core facilitated this sequential coupling, enabling the creation of both homogeneous and heterogeneous segmented fibrils. This work underscores how LCBCP self-assembly can be directed toward highly ordered, multi-level nanostructures with tunable dimensions and rigidity [75].

**Figure 2:**
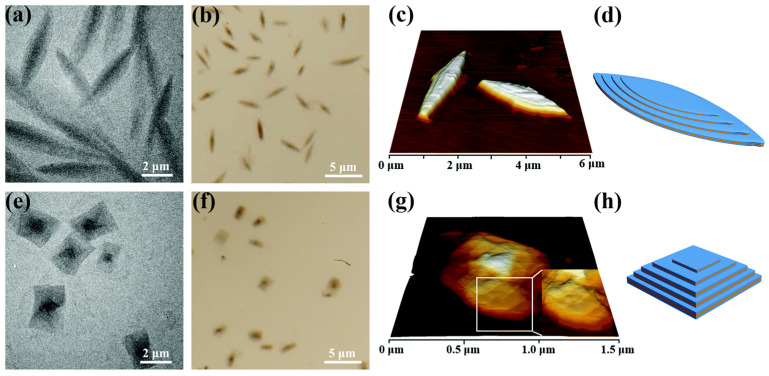
TEM (**a**), OM (**b**), and 3D AFM (Atomic Force Microscopy) (**c**) images of stacked leaf-shaped platelets self-assembled from PEG45-*b*-PAzoMA180 upon natural cooling within 1 h. TEM (**e**), OM (**f**), and 3D AFM (**g**) images of stacked rectangular platelets self-assembled from PEG45-*b*-PAzoMA180 upon gradient cooling within 7 h. (**d**,**h**) are the corresponding schematic diagrams. The images were reproduced with permission from Ref. [76].

Beyond microphase separation control, recent studies have pushed toward the hierarchical assembly of complex, multi-dimensional architectures. Hierarchical assembly of complex nanostructures is critical for creating advanced artificial systems, yet controlling 2D and 3D morphologies with LCBCPs remains difficult. Sun et al. [76] demonstrated that an azobenzene-containing liquid-crystalline diblock copolymer, PEG-*b*-PAzoMA, undergoes a rich sequence of morphological transformations during thermal annealing in ethanol. At moderate concentrations, the copolymer initially forms stacked leaf-shaped platelets (Figure 2). With increasing concentration, these evolve into cross-shaped and ultimately flower-like hierarchical platelets, a progression indicative of concentration-mediated nucleation and aggregation control. Moreover, slow cooling promotes a transition from 2D leaf platelets into 3D rectangular platelets, revealing that annealing kinetics critically influence domain geometry. Adding an extra dimension to this self-assembly, the photoactive azobenzene cores enable UV-induced trans–cis isomerization, which in turn triggers reversible morphological reconfiguration of the platelet structures. Together, these capabilities illustrate how tuning solution conditions and thermal protocol, in combination with molecular photo-responsiveness, enables the fabrication of hierarchically structured, stimuli-adaptive nanomaterials, a promising foundation for applications in templating, sensing, photonic modulation, and beyond.

### 2.2. Light–Matter Interaction and Photonic Functionality in LCBCPs

Beyond structural control for nanoscale templating and hierarchical assembly, the self-assembly principles of LCBCPs can also be exploited to manipulate light–matter interactions. This capability makes them highly attractive for photonic applications, where their ordered morphologies and tunable optical properties enable the formation of photonic bandgap structures. Such structures underpin advanced devices including optical filters, waveguides, and tunable lasers [41,77].

Recent research has focused on exploiting this optical tunability through advanced molecular designs and self-assembly pathways. In their 2024 study, Yang et al. [78] synthesized an LCBCP featuring a discotic triphenylene (HAT) mesogen block and explored its solution-state self-assembly driven by strong π–π stacking interactions. They reported a profound sequence of morphological transformations: starting with spherical micelles, the LCBCP evolves through worm-like and tubular structures, then forms vesicular aggregates and ultimately assembles into thin fibrillar networks. These transitions occur spontaneously over extended aging periods, reflecting a subtle but powerful LC ordering effect. Remarkably, the introduction of small-molecule dopants shifts the self-assembly mechanism from intramolecular rearrangement to a nucleation-growth mode, enabling controlled formation of uniform fibrils and organo-gels via end-to-end coupling. This tunable assembly underscores the potential of discotic LCBCPs for crafting optoelectronic nanofibrils with organized π–stacked architectures, valuable in photonic and electronic applications [78].

**Figure 3:**
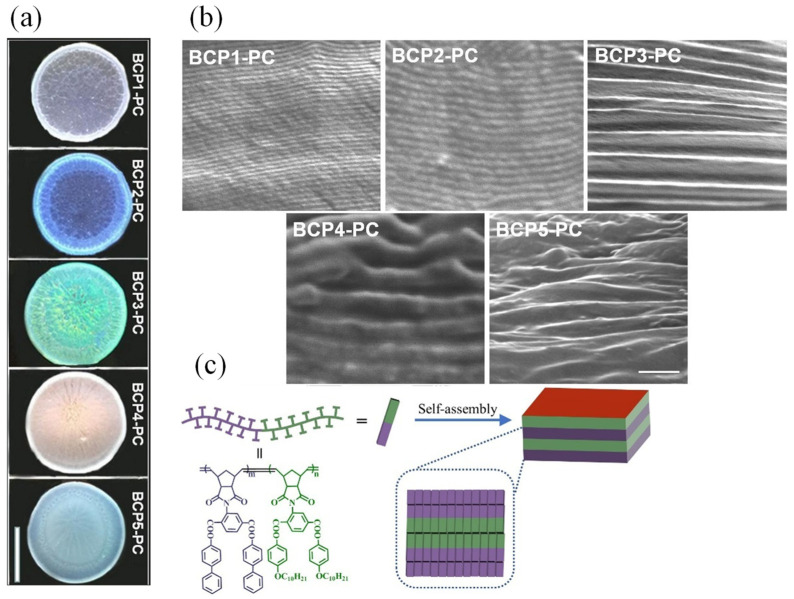
(**a**) Optical photographs (scale bar = 1 cm); (**b**) cross-sectional SEM images (scale bar = 1 μm); (**c**) schematic illustration of 1D PC thin films prepared by the self-assembly of mesogen-jacketed liquid crystals like PBNM-*b*-PDNM BCPs (adapted with permission from Ref. [79]).

While discotic LCBCPs demonstrate the power of π-stacking-driven morphologies, other approaches leverage lamellar architectures to achieve photonic functionality. Ji et al. [79] reported mesogens-jacketed liquid-crystal-like block copolymers (MJ-LCBCPs) that self-assemble into lamellar structures functioning as 1D photonic crystal (PC) thin films (see Figure 3). The mesogens-jacketed rod block, based on benzene terephthalate derivatives, provided a high refractive index, while the coil block maintained processability. Spin-coated films exhibited well-ordered lamellae, confirmed by SAXS and cross-sectional SEM, producing strong Bragg reflections tunable from the UV to near-IR by varying the degree of polymerization. This molecular-weight-dependent periodicity allowed precise control over the stop-band position, enabling color tuning without post-processing. The modular synthesis and robust LC ordering of MJ-LCBCPs highlight their potential for optical coatings, sensors, and photonic device applications.

**Figure 4:**
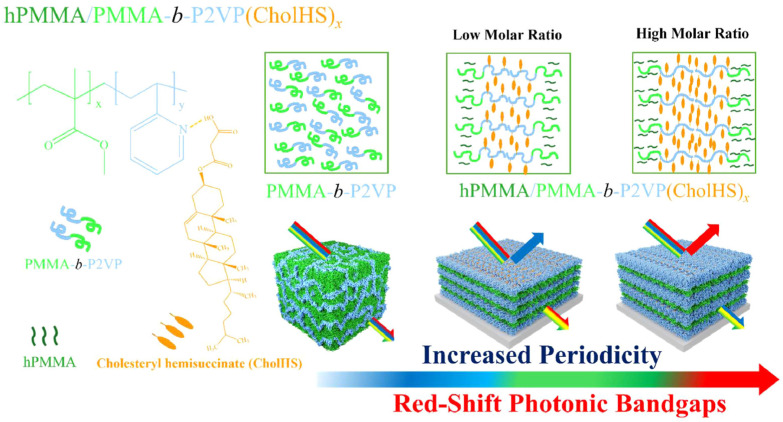
Schematic representation of the rapid fabrication of hPMMA/PMMA-*b*-P2VP(CholHS)_x_ supramolecular assemblies, in which the lamellar periodicity and microstructural orientation are simultaneously controlled. The periodicity, governed by the stretching of the P2VP chains in the host block copolymer, is tuned through hydrogen bonding with guest CholHS molecules. By varying the amount of CholHS, both the lamellar spacing and the resulting photonic bandgap can be precisely adjusted. Concurrently, the liquid-crystalline nature of CholHS promotes the alignment of parallel lamellar domains, enabling well-ordered photonic structures. The schematic drawing was reproduced with permission from Ref. [80].

Expanding on this lamellar-based photonic design, recent research has shifted toward scalable, non-labor-intensive processing routes that preserve or even improve structural order in liquid-crystalline block copolymer (LCBCP) systems. In 2025, AboElsood et al. [80] reported a rapid solution-casting strategy in which coincident augmentation (growth of lamellar domain size) and alignment occur synergistically during solvent evaporation (Figure 4). This method exploits the interplay between solvent–polymer interactions and evaporation-induced flow fields to promote both the lateral expansion of lamellae and the in-plane orientation of their stacking direction. Remarkably, the films, comprising solid-state block copolymer photonic crystals, exhibit intense, angle-dependent structural coloration with composition-tunable reflection peaks spanning the visible spectrum. The entire fabrication process is completed within minutes, requiring no post-processing such as thermal annealing or shear alignment, while still achieving optical performance comparable to more energy- and time-intensive methods. The approach is also compatible with large-area casting, indicating strong potential for scalable manufacturing of structurally colored films. Collectively, such advances demonstrate how careful molecular design of LCBCP lamellae, combined with process-driven structural control, can deliver precisely tuned photonic responses for applications in optical coatings, sensors, anti-counterfeiting devices, and display technologies.

### 2.3. Biomedical Applications

LCBCPs can be engineered for biomedical use by incorporating biocompatible segments, such as polyethylene glycol or cholesterol, while retaining mesogen-driven order and stimuli-responsiveness, enabling multifunctional carriers and scaffolds with precisely tunable structure–function relationships [81,82,83].

One promising direction in this context is the development of biomimetic scaffolds that replicate the structural cues of native tissues. LCBCs can form scaffolds with ordered nanostructures that mimic biological tissues, supporting cell growth and differentiation. Prévôt et al. [82] reported LCBCP-derived foams prepared by crosslinking star block copolymers bearing cholesteryl side groups, producing interconnected, brain-like porous scaffolds with SmA ordering. These scaffolds supported high cell viability and promoted contact guidance along anisotropic LC cues; their mechanical properties and biodegradation profile were tailored to mimic soft neural tissue [82].

**Figure 5:**
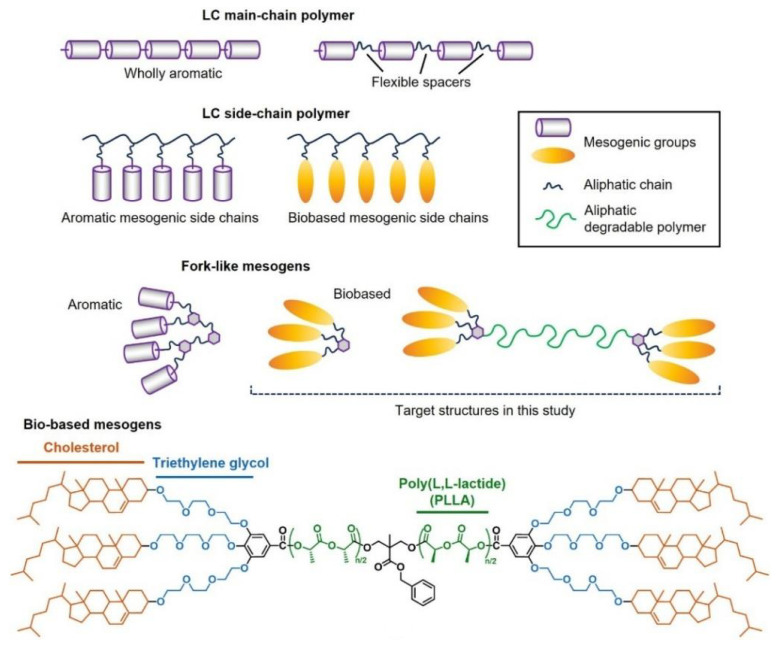
Molecular design for liquid-crystalline (LC) poly(L,L-lactide) derivatives in comparison with those of the conventional and previous LC polymers (reproduced with permission from Ref. [84]).

Expanding beyond specific scaffold systems, the broader family of LC polymers offers valuable architectural insights for biomedical LCBCP design. In 2025, as illustrated in Figure 5 of the work by Gong et al. [84], LCPs encompass diverse architectures, wholly aromatic main-chain systems, aromatic/aliphatic multiblock copolymers, and thermotropic side-chain polymers, each exploiting mesogen self-assembly to generate ordered nanostructures with dynamic properties. These structural principles translate directly to LCBCPs designed for biomedical use, where careful placement of mesogenic units within main-chain, multiblock, or side-chain segments allows simultaneous control over mechanical performance, optical response, and surface interactions. For example, introducing aromatic mesogens into the block architecture can provide mechanical anisotropy for load-bearing tissue scaffolds, while sidechain mesogens in amphiphilic LCBCPs enable stimuli-responsive drug release. Similarly, multiblock LCBCPs combining rigid mesogenic domains with soft biodegradable segments can produce elastomeric, bioadaptable materials that guide cell alignment and dynamically adapt to their environment [84].

In addition to structural scaffolds, LCBCPs have shown remarkable promise in biosensing technologies. In the realm of biosensing, Omer et al. [85] developed a liquid crystal-based biosensor incorporating a block copolymer. The system creates a monolayer at the interface between a nematic LC and water, transferred via a Langmuir–Blodgett process. When exposed to positively charged proteins below their isoelectric points, the electrostatic interaction at the interface induces a change in the LC orientation, from homeotropic (molecules perpendicular to the surface) to planar, visible under polarized optical microscopy. Detection sensitivities ranged from 0.02 to 0.08 wt% for biomarkers such as hemoglobin and chymotrypsinogen [85].

Similarly, the drug delivery field has benefited from the unique structural versatility of LCBCPs. As shown in Figure 5, Jia et al. [86] developed cholesterol-containing LCBCPs with reduction-cleavable disulfide linkages that co-assembled with croconaine into photothermal–chemo co-micelles. These nanocarriers achieved NIR-triggered heating, glutathione-responsive doxorubicin release, real-time fluorescence/photothermal imaging, and significant tumor suppression in mice, demonstrating how LC order combined with block architecture enhances loading density, stability, and stimulus-responsiveness.

Light-responsive LCBCPs have further expanded biomedical functionality by enabling non-invasive, reversible control over nanostructure and release function. This concept was extended by Wen et al. [87] that reported the synthesis of photo-responsive azobenzene-containing LCBCPs, PDMAEMA-*b*-PMAAz, via polymerization-induced hierarchical self-assembly (PIHSA) using RAFT dispersion polymerization. The process yielded nanoparticles with well-defined internal liquid crystalline ordering and diverse morphologies, including spheres, worms, and fibers, determined by block composition and assembly conditions. The azobenzene mesogens within the LC block enabled reversible, light-induced morphological transitions through trans–cis isomerization under alternating UV and visible light [87].

**Figure 6:**
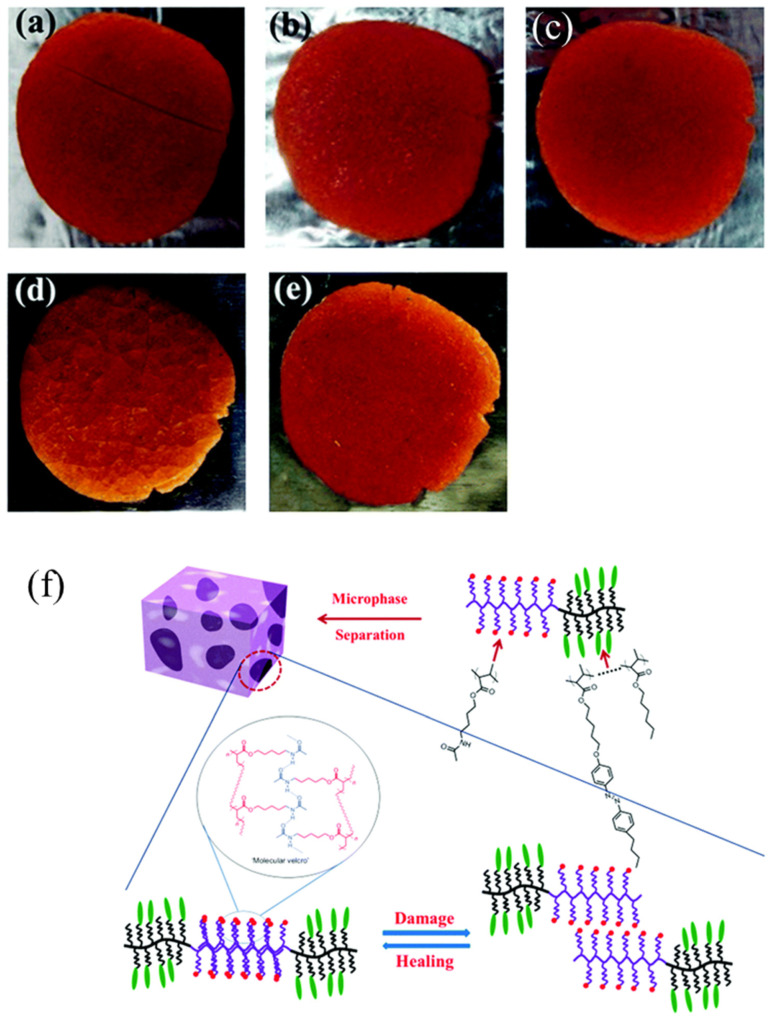
Self-healing test of BCP-3: (**a**) cut at room temperature; (**b**) warming at 50 °C for 30 min; (**c**) warming at 50 °C for 60 min; (**d**) brittle fracture in liquid nitrogen; (**e**) warming at 50 °C for 3 h. (**f**) The aggregation morphology and the self-healing process of the BCPs. The figure was reproduced with permission from Ref. [88].

Complementing these functional nanocarriers, dynamic and adaptive LCBCP-based materials have been developed for tissue engineering. Yan et al. [88] developed supramolecular LC elastomers (LCEs) from block copolymers combining a side-chain LC polymer with hydrogen-bonding soft segments. The hydrogen bonds enable autonomous self-healing at room temperature (see Figure 6), while the LC phase enhances mechanical robustness and structural order, highlighting the potential for dynamic, self-repairing biomaterials in tissue engineering.

Together, these examples illustrate how mesophase ordering within microphase-separated domains can be strategically harnessed to guide cell alignment, regulate drug encapsulation and stimulus-triggered release, and integrate diagnostic and therapeutic capabilities within a single, multifunctional biomedical platform.

### 2.4. Energy Storage and Ion Transport

While initially studied in biomedical systems, attention has also turned to electronic systems, in which the ordered morphologies and dynamic responsiveness of LCBCPs can also be exploited in energy-related applications, with precise molecular organization playing a pivotal role in charge and ion transport. Recent advances in polymers have significantly enhanced molecular solar thermal fuels by improving energy storage capacity, half-life, and spectral absorption through strategies like molecular design and template self-assembly. These advances—spanning composites, linear, dendrimer, and hybrid polymer architectures—underscore LCBCPs as capable candidates for efficient, stimuli-responsive solar thermal energy materials. Their self-assembled nanostructure enhances charge transport, while their flexibility supports applications in wearable and foldable devices [89,90].

A key advantage of LCBCPs in this domain lies in their ability to combine anisotropic mesogen ordering with microphase-separated nanodomains to create efficient transport pathways. The anisotropic ordering of mesogenic units in LCBCPs, combined with their microphase-separated nanodomains, generates continuous, well-defined channels for directional ion transport. In smectic or columnar phases, aligned mesogenic segments form one-dimensional ion-conductive pathways, while flexible blocks provide mechanical stability and electrolyte compatibility. Such architectures have shown enhanced performance in lithium-ion batteries, solid-state electrolytes, and supercapacitors, where anisotropic ionic conductivities surpass those of amorphous polymers [90].

**Figure 7:**
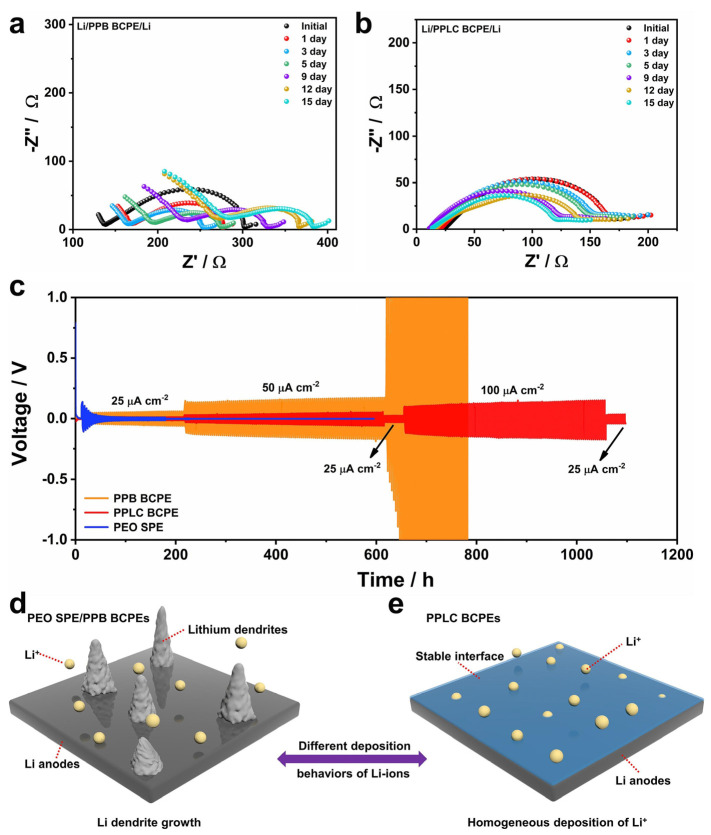
(**a**) The changes of interface impedance of the Li/PPB BCPEs/Li symmetrical batteries for different aging times at 60 °C. (**b**) The interface impedance of the Li/PPLC BCPEs/Li symmetrical batteries with the change of storage time at 60 °C. (**c**) Voltage profiles of Li/PPB BCPEs/Li, Li/PPLC BCPEs/Li, and Li/PEO SPE/Li symmetrical batteries during galvanostatic lithium stripping/plating experiments at different current densities of 25, 50, and 100 µA·cm^−2^. (**d**,**e**) Schematic illustration of the contrasting Li+ deposition behaviors in Li/PEO SPE/Li, Li/PPB BCPEs/Li (**d**), and Li/PPLC BCPEs/Li (**e**) batteries. The figure was reproduced with permission from Ref. [91].

Recent material innovations demonstrate how targeted molecular design can further boost electrochemical performance. For instance, Zeng et al. [91] developed a boron-containing LC-multiblock copolymer electrolyte PPB-BCPE in which aligned LC domains form continuous Li^+^ channels, while electron-deficient boron atoms immobilize TFSI^−^ anions to enhance ion transport. Figure 7 shows that Li/PPLC-BCPE/Li cells exhibit low, stable interfacial resistance during storage at 60 °C, unlike PPB BCPEs with irregular resistance due to poor electrode contact. In stripping/plating tests, PPLC-BCPE cells cycle for >1000 h at 25–100 µA·cm^−2^ with low overpotentials, whereas PEO and PPB BCPEs fail due to dendrite growth. SEM confirms smooth, dendrite-free Li surfaces for PPLC-BCPEs, highlighting how LC ordering and boron chemistry synergistically stabilize interfaces and suppress dendrites in solid-state batteries [91].

**Figure 8:**
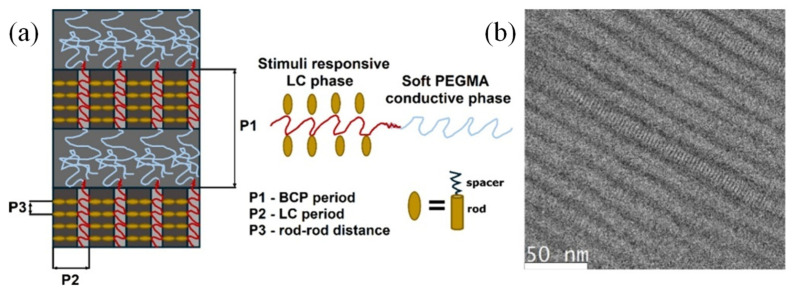
Hierarchical morphology of LC-containing BCPs and BCPEs: (**a**) scheme depicting the proposed structure, where LCs anchor homogeneously with respect to the BCP inter-material dividing surface, resulting in the perpendicular orientation of the smectic layer; (**b**) TEM micrograph depicting the lam-in-LAM microstructure of S1-Salt/DC with an LC layer spacing P2 of 3 nm. The figure was reproduced with permission from Ref. [92].

Processing strategies have also proven critical for optimizing ionic conductivity in LCBCP electrolytes. Gohy et al. [92] demonstrated that the synthesis of MALC-*b*-(EGMA-co-GM) enabled the formation of a smectic mesophase over a wide temperature range, both with and without lithium salt (see Figure 8). Their work highlighted that applying electric fields during solvent casting significantly improved the ionic conductivity of the resulting polymer electrolytes, achieving conductivities nearly an order of magnitude higher than films cast without an electric field, due to enhanced phase separation and longer-range ordering. Furthermore, they observed that DC electric fields promoted larger grain sizes and more extensive ordering compared to AC fields and that the smectic layers oriented perpendicularly to the applied field, a phenomenon attributed to dielectric contrast effects between the block copolymer components rather than the liquid crystalline mesogens themselves. These findings provide valuable insights into the design of block copolymer electrolytes with tailored morphologies for efficient lithium-ion transport [92].

**Figure 9:**
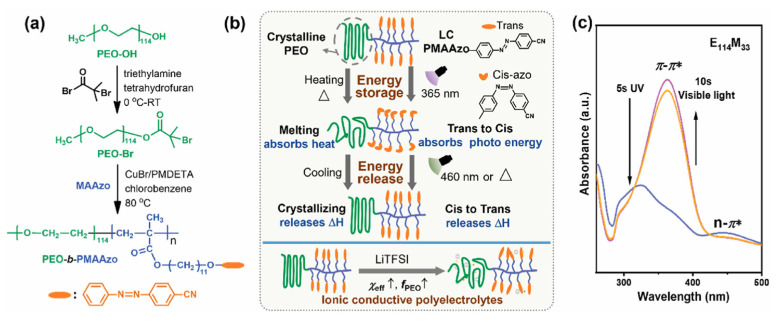
(**a**) Synthesis route of PEO-*b*-PMAAzo block copolymers. (**b**) Schematic illustration of the energy and release process during the melting-crystallization of PEO and the photoisomerization of the Azo group under 365 nm UV light and 460 nm visible light irradiation, as well as the lithium salt solvation in the BCPs, which induce increases in χ and fPEO values and enable ionic conductivity. (**c**) UV–Vis spectra of E114M33 in solutions measured before and after 365 nm UV light irradiation for 5 s and then after 460 nm visible light irradiation for 10s. The figure was reproduced with permission from Ref. [93].

Beyond purely ionic systems, LCBCPs can integrate multiple energy storage mechanisms within a single material platform. Figure 9 shows that Sun et al. [93] synthesized a series of biphasic, phase-change azobenzene LCBCPs (PEO-*b*-PMAAzo) with remarkable energy storage capabilities. In this system, the PEO block stores energy via crystallization–melting transitions, while the azobenzene-containing PMAAzo block enables solar–chemical–thermal energy conversion through reversible conformational changes. Coordination of Li^+^ ions with both the PEO backbone and azobenzene units further facilitates biphasic ion conduction. By tuning block composition, applying UV irradiation, and varying lithium salt concentration, they achieved precise control over thermal behavior and self-assembly, generating thermally stable nanostructures (LAM, GYR, Fddd, HEX, and HPL) that are particularly advantageous for ion transport in electrochemical devices [93].

### 2.5. Stimuli-Responsive LCBCPs

LCBCPs can be designed to undergo stimuli-responsive changes, such as altering wettability, adhesion, or optical properties, triggered by external cues like light, temperature, electric or magnetic fields, and solvent exposure. This responsiveness enables applications across anti-fouling surfaces, self-cleaning coatings, and adaptive optical films for microfluidic or smart window technologies. For example, azobenzene-containing LCBCPs have been shown to undergo photo-induced reconfiguration of nanoscale morphology, such as from hemimicelles to chain-like structures upon UV exposure, demonstrating how light can precisely tune surface or film properties [94].

**Figure 10:**
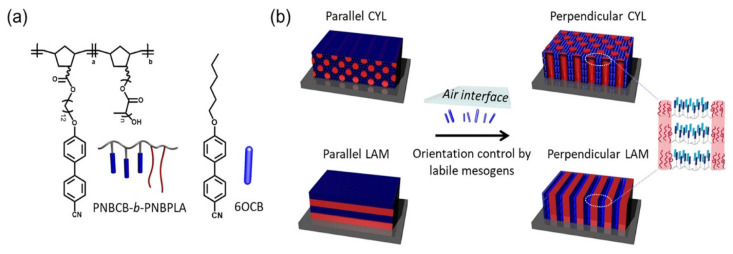
(**a**) Chemical structures of PNBCB-*b*-PNBPLA and 6OCB. (**b**) Schematic illustration for orientation control of CYL and LAM structures by labile mesogen addition. The labile mesogens adopt planar or homogeneous anchoring at the IMDS. The figure was reproduced with permission from Ref. [95].

In addition to light-driven surface modulation, mesogen anchoring effects can be exploited to control microdomain orientation in thin films. Osuji et al. [95] investigated how the morphology of LCBCP thin films was controlled by the anchoring behavior of free, non-bonded (labile) mesogens at the film’s surface (see Figure 10). They found that adding mesogens induced a change in the orientation of LCBCP domains (cylinders or lamellae) from parallel to perpendicular relative to the film surface. This occurred because the labile mesogens adopted homeotropic (perpendicular) anchoring at the air interface, which, combined with homogeneous anchoring at the internal BCP interfaces, led to the perpendicular alignment of the BCP superstructure. Moreover, by systematically varying mesogen concentration, film thickness, and annealing conditions, they showed that the labile mesogens co-assembled with backbone-bound mesogens and influenced film morphology. They also demonstrated the formation of perpendicular BCP structures on topographically patterned substrates. Their work highlighted the importance of mesogen anchoring and steric constraints in controlling LCBCP film morphology and proposed design principles for mesogenic additives to engineer such morphologies [95].

**Figure 11:**
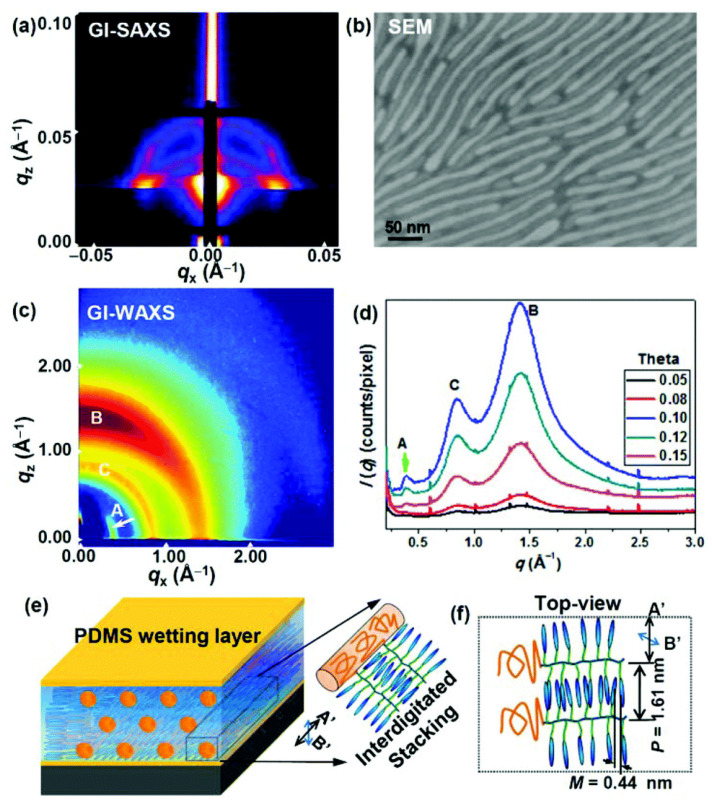
(**a**) GISAXS pattern with an incident angle of 0.15° of the 140 nm thick BCP film after annealing at 120 °C; (**b**) a representative SEM image of oxidized PDMS cylindrical nanopatterns formed in the thin film; (**c**) 2D GIWAXS pattern with an incident angle of 0.15°; (**d**) the 1D GIWAXS profiles with an incident angle from 0.05° to 0.15°; (**e**,**f**) schematic illustrations of the in-plane cylinder with LC mesogens in a smectic LC phase, where the mesogen π–π interaction direction is perpendicular to the smectic layer direction. The figure was reproduced with permission from Ref. [96].

Temperature-dependent order–order transitions present another powerful mechanism for dynamic structure control. Liao et al. [96] investigated a high-interaction-parameter, silicon-containing side-chain LCBCP, PDMS-b-P(4CNB11C)MA (poly(dimethylsiloxane)-block-poly(4-cyanobiphenyl-based LC methacrylate)), with a PDMS volume fraction of ~27%. In bulk, the BCP self-assembled into sub-20 nm hexagonally packed (HEX) PDMS cylinders within a smectic LC matrix (smectic layer period: ~1.61 nm). Notably, the HEX structure persisted even after the LC block transitioned to isotropic (~120 °C). As Figure 11, in 140 nm thin films, PDMS cylinders lay parallel to the substrate; upon heating to ~160 °C, an order-to-order transition occurred, transforming the morphology from HEX cylinders to body-centered cubic (BCC) spheres. This dynamic behavior was captured in real time using in situ GISAXS (Grazing-Incidence Small-Angle X-ray Scattering) and GIWAXS (Grazing-Incidence Wide-Angle X-ray Scattering), both performed at National Synchrotron Light Source II at Brookhaven National Laboratory, and ex situ SEM and AFM elucidated the transient pathway between structures. GI-WAXS analysis revealed a sharp reflection from the smectic layer spacing and a broad arc from π–π mesogen stacking. These features persisted up to ~120 °C, indicating stable LC order even into the isotropic phase, but weakened near 160 °C, coinciding with the HEX → BCC morphology transition [96].

Coupling phase transitions with external fields can further amplify microdomain alignment. Gopinadhan et al. [97] demonstrated that LCBCPs undergoing a nematic-to-smectic-A (N→SmA) transition exhibit a dramatic increase in microdomain alignment when cooled under an applied magnetic field. While the nematic phase yielded weak alignment, entering the smectic-A phase triggered a strong orientational coupling between LC layering and block copolymer microdomains, yielding tightly aligned nanostructured arrays. In situ SAXS/WAXS during magnetic-field-assisted cooling of LCBCPs showed weak microdomain alignment in the nematic phase, but a sharp increase at the N–SmA transition. WAXS detected the onset of smectic layering, revealing strong coupling between LC translational order and block copolymer microdomain orientation. This finding underscores the potential of harnessing LC phase transitions for field-directed thermal-induced LCBCPs [97].

**Figure 12:**
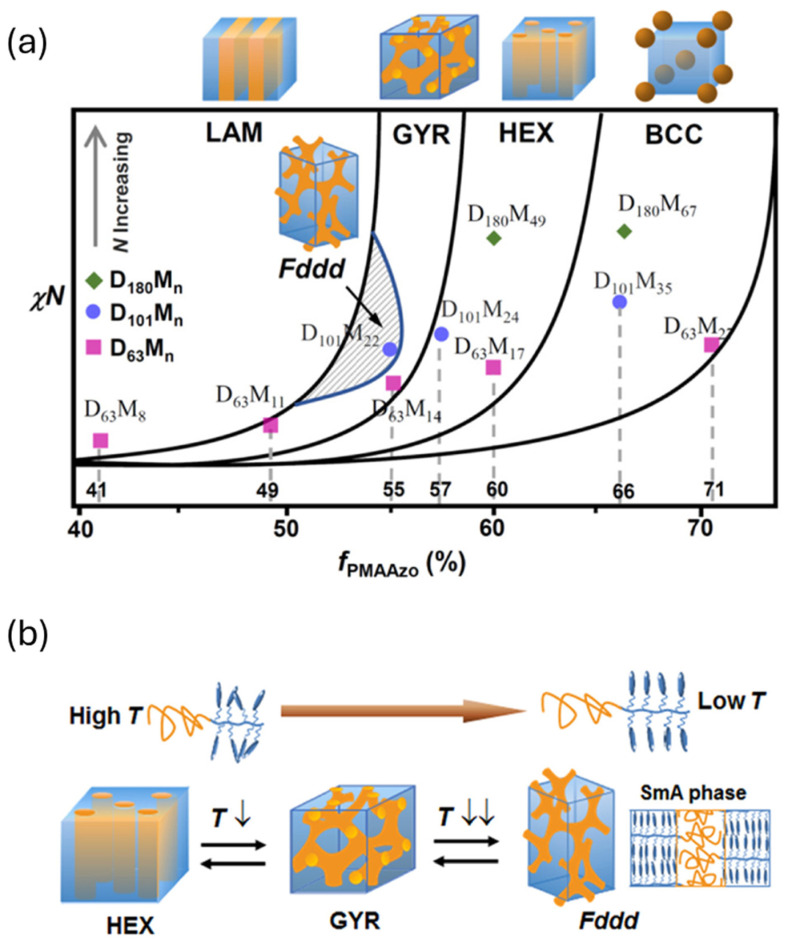
(**a**) Schematic illustration of the phase diagram, χN vs. fPMAAzo, of the PDMS-*b*-PMAAzo BCPs with different total molecular weights. (**b**) Schematic illustration of the HEX-to-GYR-to-Fddd transition as well as the LC phase transition of the PMAAzo blocks. The figure was reproduced with permission from Ref. [52].

Stimuli-responsiveness can also be combined with complex network morphologies to achieve multifunctional materials. Weng et al. [52] synthesized silicon-containing PDMS-*b*-PMAAzo LCBCPs and examined their thermally induced structure-in-structure phase transitions and thin-film assembly. Varying the PMAAzo content yielded bulk morphologies including LAM, GYR, Fddd, HEX, and BCC (referred to as lamellar, gyroid, orthorhombic, hexagonal-cylinder, and body-centered cubic phases, respectively) after annealing at 150 °C, with LC phase behavior favoring the Fddd orthorhombic network. These LCBCPs combine morphology tunability, network structure formation, and thermos/photo-induced orientation switching (see Figure 12).

Surface-engineered LCBCPs represent an additional frontier for adaptive materials. In 2025, Feng et al. [98] reported a hierarchically structured, photo-responsive azo-copolymer coating inspired by natural superwetting surfaces. The terpolymer integrates azobenzene mesogens for light-triggered molecular reorientation, catechol groups for strong substrate adhesion, and fluorinated segments for low surface energy. Dip-coating onto a micro/nanostructured copper mesh produced a surface capable of reversible wettability switching between superhydrophobic and super-hydrophilic states under UV/visible light, driven by azobenzene trans–cis isomerization within the LC domains. The hierarchical surface structure amplified this effect, enabling robust, repeatable transitions even under abrasive and chemically harsh conditions. This study exemplifies how LC polymer architectures with multifunctional components can be engineered for adaptive, durable interfaces, offering clear design parallels for future LCBCP-based smart coatings and microfluidic devices.

### 2.6. Soft Robotic Systems

Soft robotic systems represent an emerging application area where LCBCPs hold unique promise. Soft robots offer promising potential for minimally invasive medical procedures but face significant challenges in fabrication, power, and navigation [99,100,101,102,103,104,105,106,107,108,109]. Despite their similarities to biological materials, LCBCPs remain an underexplored resource for designing these types of soft robots [50,110]. Moreover, soft robotics could be considered a subset of stimuli-responsive materials, since actuation and motion rely on controlled structural changes triggered by external cues such as heat, light, or electric/magnetic fields. However, this review discusses soft robotics in a dedicated section to emphasize the unique potential of LCBCPs in creating programmable, adaptive actuators that combine nanoscale ordering with macroscopic deformation. LCBCPs offer hierarchical control that enables fine-tuning of mechanical anisotropy, responsiveness speed, and actuation amplitude.

**Figure 13:**
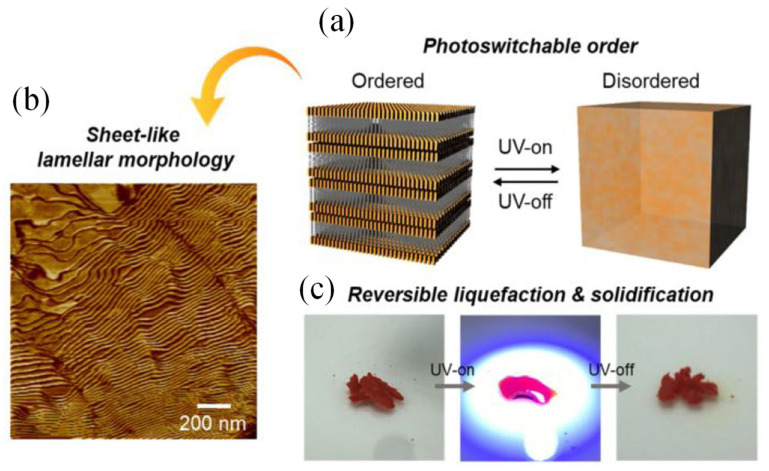
(**a**) AFM phase images of NO2-Azo12-ODMS. (**b**) Schematic illustration on the photo-switchable order. (**c**) Photographs showing switchable liquefaction and solidification. The figure was reproduced with permission from Ref. [57].

Recent work has shown how molecular design parameters can directly influence actuation kinetics and structural transitions. Lee et al. [57], in their recent study, developed Azo-based LCBCPs with rapid photo-switchable ordering transitions, revealing how end groups and carbon spacers influence self-assembly and switching kinetics. End groups and spacers modulate liquid-crystalline interactions, with a 12-carbon spacer enabling structure-in-structure organization (see Figure 13). All LCBCPs showed ordering and disordering within seconds, reordering at room temperature without heating or visible light, driven by strong electron-withdrawing groups (NO_2_, CN) and LC mesophase cooperativity. Rich polymorphism emerged, depending on illumination and thermal history, reflecting mesogen packing flexibility and triblock loop/bridge conformations [57].

**Figure 14:**
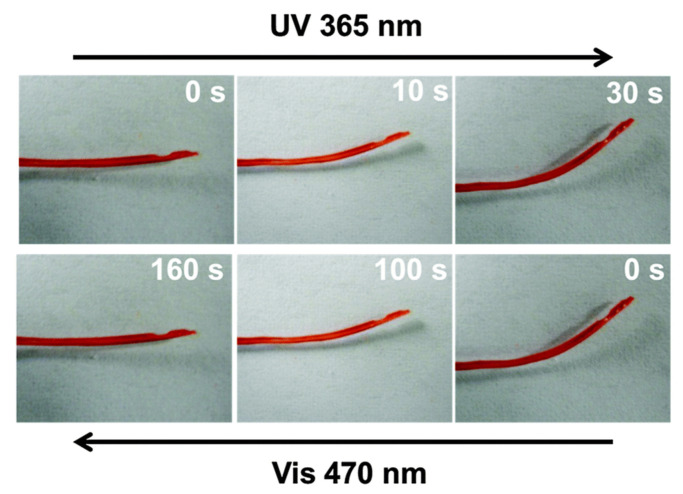
Images of photoinduced bending and unbending of the CLCP fiber upon irradiation with UV light and visible light (reproduced with permission from Ref. [111]).

Building on such photomechanical responses, LCBCPs have been integrated into mechanically robust, yet highly responsive actuator films. A representative example of LCBCPs applied in soft actuation was reported by Lv et al. [111], see Figure 14, who synthesized a reactive liquid crystalline block copolymer composed of polyethylene oxide (PEO) and an azobenzene-containing polymethacrylate block (PEO-*b*-PAZO). The material exhibited well-defined microphase separation with a lamellar morphology, combining the ion-conducting properties of PEO with the photo-responsive anisotropic ordering of the azobenzene mesogens. Light crosslinking preserved both LC alignment and block copolymer nanostructure, enabling mechanically robust, yet responsive films. Upon UV irradiation, trans–cis photoisomerization of azobenzene disrupted mesogen packing, inducing rapid and reversible bending, while visible light restored the original shape [111].

**Figure 15:**
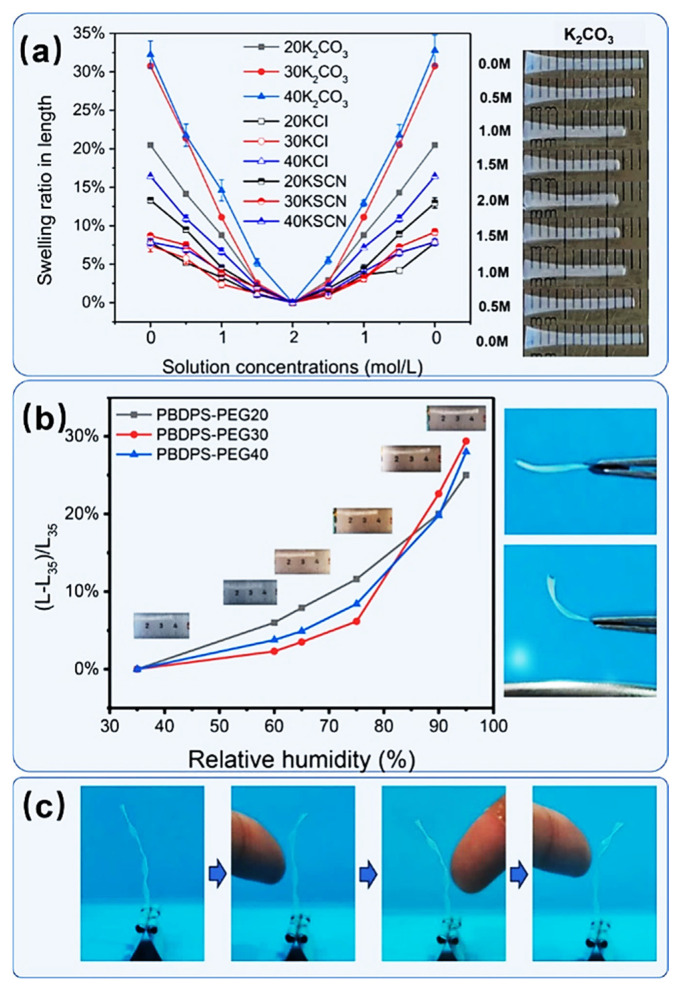
(**a**) Anisotropic swelling and deswelling of the PBDPS–PEG hydrogel actuators in salt solutions of varying concentrations; and photographs showing reversible shape changes triggered by different concentrations of K_2_CO_3_ solution. (**b**) Shape change ratio along the length of the PBDPS–PEG30 actuator at different relative humidity levels; (right) PBDPS–PEG30 actuator demonstrating a bending shape change when exposed to a bottle of water. (**c**) PBDPS–PEG30 actuator demonstrating a bending shape change triggered by proximity to a wet finger. The figure was reproduced with permission from Ref. [48].

Another exciting advancement in LCBCP-based soft actuators comes from Wang et al. [48] in 2025, where they developed stress-free, two-way shape memory copolymer actuators that leverage the synergistic properties of liquid crystalline and semicrystalline domains. Their system, designed as a block copolymer with an LC domain fused to a semicrystalline segment, demonstrates reversible, bidirectional actuation across multiple stimuli (e.g., heat, light, or chemical triggers), without requiring external mechanical bias (see Figure 15). By harnessing distinct thermal transitions in both domains, the material achieves robust shape programming and autonomous movement, effectively advancing LCBCP design toward multi-stimuli-responsive soft robotics that combine precision, resilience, and adaptability [48].

### 2.7. Additive Manufacturing Technologies

Additive manufacturing (AM) technologies represent another rapidly growing application area where liquid crystalline block copolymers (LCBCPs) can play a transformative role. AM has revolutionized modern industries by enabling the creation of complex computer-designed geometries. However, 3D-printed polymer parts often suffer from poor mechanical properties, limiting their applications. Some studies have tackled this issue by incorporating LCBCP into 3D printing.

**Figure 16:**
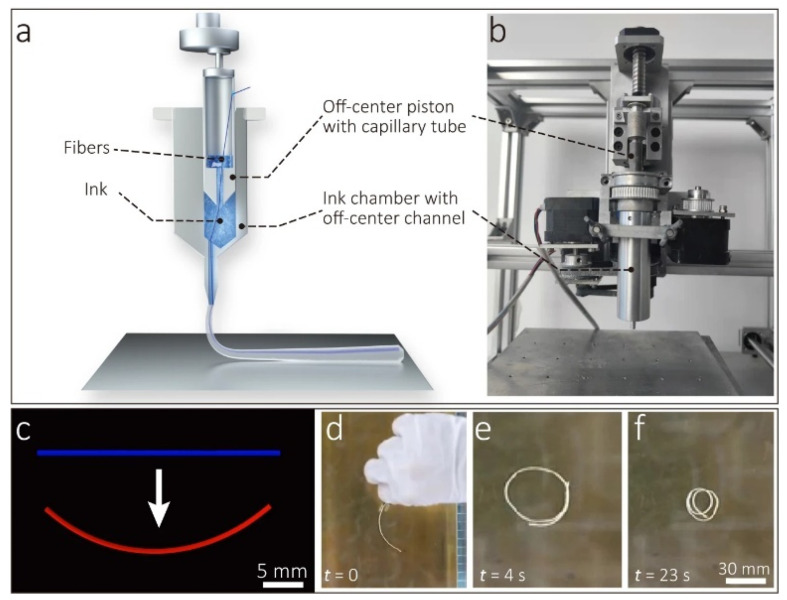
(**a**,**b**) Schematic of the equipment and simulation analysis of the thermal deformation effect of the LCE composite. (**c**) Simulation analysis of the thermal deformation effect of the LCE composite. (**d**–**f**) The deformation states of a filament at an ambient temperature of 150 °C and t = 0 s, 4 s, and 23 s. The figure was reproduced with permission from Ref. [112].

At the intersection of AM and soft robotics, recent work has demonstrated how LC-based structural design can be printed with programmable anisotropy and high strength. As shown in Figure 16, Wang et al. [112] developed a direct ink-writing 4D printing strategy for fabricating freestanding composites composed of continuous fiber-reinforced LC elastomers. By precisely controlling the off-center alignment of reinforcing fibers within the LC elastomer matrix, they achieved programmable, anisotropic deformation coupled with exceptional mechanical robustness. The printed actuators exhibited load-bearing capacities up to 2805 times their own weight and high-curvature bending when heated to 150 °C. This integration of microscale LC domain orientation with macroscale structural design offers a scalable pathway toward durable, reconfigurable, and high-performance soft robotic components for applications requiring both adaptability and mechanical strength [112].

## 3. Challenges, Limitations and Future Directions

Despite their versatility, LCBCPs face persistent challenges in scalability, cost, and precise phase control. Synthesizing high-molecular-weight LCBCPs with consistent mesogenic alignment is inherently complex, and large-scale production remains expensive. Stability under varying environmental conditions, such as temperature, humidity, and mechanical stress, must be ensured for reliable real-world performance. Device integration poses another hurdle, as LCBCPs must be compatible with existing manufacturing workflows without losing their hierarchical ordering or stimuli-responsiveness [113,114,115,116].

Addressing these limitations will require coordinated advances in synthesis, processing, and material design. Future research should prioritize scalable synthesis strategies, such as cost-effective controlled polymerizations, improved mesogen alignment methods, and post-processing protocols that preserve phase order. Computational modeling offers a powerful tool to predict LCBCP phase behavior and optimize molecular designs before synthesis. Hybrid composites, combining LCBCPs with nanoparticles, conductive fillers, or bio-derived components, could further expand their utility in electronics, energy storage, and biomedical systems.

One particularly underexplored area is the integration of LCBCPs into advanced additive manufacturing workflows. Despite the extensive exploration of AM with liquid crystalline polymers and elastomers [117,118,119,120,121,122,123], the direct integration of LCBCPs into 3D printing platforms remains virtually unexplored. Given their intrinsic hierarchical self-assembly, anisotropic ordering, and stimuli-responsive properties, LCBCPs hold exceptional promise for fabricating complex architectures with programmable mechanical, optical, and actuation functions. Incorporating LCBCPs into advanced AM techniques represents a fertile and largely untapped research area where material design, processing optimization, and structural programming could converge to unlock new capabilities in printed functional materials.

Designing covalent LC architectures could further enhance structural stability during printing and end-use performance. Building on this approach, future research in 3D printing could replace the physically blended small-molecule LC (5CB) in PR-5CB systems [119] with covalently bound LC segments, such as in LC-containing block copolymers or LC side-chain polymers. In particular, LC semicrystalline block copolymers could offer a synergistic combination of properties: the semicrystalline block providing stiffness, thermal stability, and shape retention, while the LC block imparts anisotropic ordering and stimuli-responsiveness. This covalent architecture would prevent phase separation, ensure stable LC alignment during printing, and enable the design of printed parts with long-term structural integrity, tunable mechanical anisotropy, and programmable functional responses.

Inspiration from bio-inspired design strategies offers another promising pathway. Incorporating bioinspired hierarchical design and 3D printing strategies, as demonstrated in recent composite organo-hydrogels [124], could enable LCBCPs with enhanced mechanical robustness and customizable architectures for advanced photonic, biomedical, and soft robotic applications.

Concepts from supramolecular actuator design may also be translated into LCBCP platforms. Yu et al. [125] fabricated a photo-responsive mechanical actuator through intermolecular hydrogen−bond interactions. In this supramolecular network, UV light triggers trans–cis photoisomerization of the azobenzene moieties, generating rapid and reversible bending motions. The actuator demonstrated impressive mechanical performance, capable of lifting loads up to 200 times its own mass, highlighting the efficiency of light-to-mechanical energy conversion in H-bonded architectures. Although this system is not a block copolymer, the design concept readily translates to LCBCPs: embedding azobenzene mesogens in one block and engineering complementary H-bond donor/acceptor groups in the other could yield fully BCP-based, light-driven actuators with tunable mechanical anisotropy, rapid response, and high actuation force, traits ideal for untethered soft robotic devices.

Overcoming characterization barriers is equally critical for advancing LCBCP research and other complex materials [126,127,128,129]. Characterizing the internal structure and chain behavior of LCBCP cylindrical micelles is notoriously difficult. Traditional techniques such as TEM, SAXS, and DSC can reveal morphology, phase transitions, and thermal behavior, but they often require complex sample preparation, provide indirect information about molecular packing and may not capture subtle chain conformational changes within the micelle core and corona. Furthermore, detecting structural parameters like the critical micellization concentration (CMC) and LC phase transitions inside micellar cores with high accuracy remains a significant barrier, especially for systems where the LC domain’s ordering strongly influences functionality [42].

Innovative in situ and non-invasive techniques can help overcome these obstacles. In 2025, He et al. [130] addressed these limitations by incorporating aggregation-induced emission (AIE) moieties into carefully designed LCBCPs. The AIE effect, where fluorescence intensifies upon aggregation, enabled direct, sensitive, and non-invasive monitoring of micelle formation and internal ordering. By positioning the AIE unit in either the LC core-forming block or the corona-forming block, they could selectively probe different micellar regions. This approach allowed accurate determination of CMC and core LC phase transition temperatures and real-time monitoring of self-assembly without disruptive sample handling.

Although LCBCPs are still at an early research stage, their unique properties suggest strong potential for future industrial translation. Unlike conventional LCPs, which are already commercialized in fiber and engineering plastics (such as Vectra), LCBCPs have not yet reached the market. Bridging this gap will require advances in scalable synthesis, processing, and application-driven design. We anticipate that the coming decade will see LCBCPs moving closer to practical use, with opportunities in many diverse applications.

## 4. Conclusions

Liquid crystalline block copolymers (LCBCPs) uniquely integrate microphase separation with mesogen-directed anisotropy, enabling hierarchical ordering across multiple length scales. This structural duality underpins their demonstrated utility in nanofabrication, photonics, biomedicine, energy storage, adaptive interfaces, soft robotics, and additive manufacturing. Recent advances highlight the power of molecular precision via tailored mesogen chemistry, blocks architecture, and processing pathways, to program coupled optical, mechanical, and stimuli-responsive functions. However, translating these laboratory successes into scalable, device-ready technologies remains constrained by challenges in high-molecular weight synthesis, mesogen alignment control, environmental stability, and manufacturing integration. Addressing these bottlenecks through synergistic advances in covalent LC architectures, bio-inspired structural motifs, computationally guided design, and high-resolution AM could unlock LCBCPs as a foundational materials platform for multifunctional, reconfigurable systems that bridge molecular order with macroscopic function.

## Data Availability

No new data were created or analyzed in this study. The GA and Scheme 1 are plotted by Authors.
